# The human RECQ1 helicase is highly expressed in glioblastoma and plays an important role in tumor cell proliferation

**DOI:** 10.1186/1476-4598-10-83

**Published:** 2011-07-13

**Authors:** Ramiro Mendoza-Maldonado, Valentina Faoro, Sailesh Bajpai, Matteo Berti, Federico Odreman, Marco Vindigni, Tamara Ius, Abdollah Ghasemian, Serena Bonin, Miran Skrap, Giorgio Stanta, Alessandro Vindigni

**Affiliations:** 1 International Centre for Genetic Engineering and Biotechnology Padriciano 99, 34149 Trieste, Italy; 2 ACADEM Department, Cattinara Hospital, Surgical Pathology Building, Strada di Fiume 447, 34149 Trieste, Italy; 3 Neurosurgery Unit, Azienda Ospedaliero-Universitaria, Piazzale Santa Maria della Misericordia 15, 33100 Udine, Italy; 4 Department of Biochemistry and Molecular Biology, Saint Louis University School of Medicine, St Louis, MO 63104, USA; 5 Sincrotrone Trieste S.C.p.A. SS 14 - km 163,5 - AREA Science Park, 34149 Basovizza, Trieste, Italy; 6 Department of Pharmaceutical Biotechnology, Faculty of Pharmacy, Shiraz University of Medical Sciences, Shiraz, Iran

## Abstract

**Background:**

RecQ helicases play an essential role in the maintenance of genome stability. In humans, loss of RecQ helicase function is linked with predisposition to cancer and/or premature ageing. Current data show that the specific depletion of the human RECQ1 helicase leads to mitotic catastrophe in cancer cells and inhibition of tumor growth in mice.

**Results:**

Here, we show that RECQ1 is highly expressed in various types of solid tumors. However, only in the case of brain gliomas, the high expression of RECQ1 in glioblastoma tissues is paralleled by a lower expression in the control samples due to the poor expression of RECQ1 in non-dividing tissues. This conclusion is validated by immunohistochemical analysis of a tissue microarray containing 63 primary glioblastomas and 19 perilesional tissue samples, as control. We also show that acute depletion of RECQ1 by RNAi results in a significant reduction of cellular proliferation, perturbation of S-phase progression, and spontaneous γ-H2AX foci formation in T98G and U-87 glioblastoma cells. Moreover, RECQ1 depleted T98G and U-87 cells are hypersensitive to HU or temozolomide treatment.

**Conclusions:**

Collectively, these results indicate that RECQ1 has a unique and important role in the maintenance of genome integrity. Our results also suggest that RECQ1 might represent a new suitable target for anti cancer therapies aimed to arrest cell proliferation in brain gliomas.

## Background

RecQ helicases are a ubiquitous family of DNA unwinding enzymes involved in the maintenance of chromosome stability. Five members of the RecQ family have been found in human cells: BLM, RECQ1 (also known as RECQL or RECQL1), RECQ4, RECQ5, and WRN [1-3]. Mutations in the genes of three human RecQ family members are linked to defined genetic disorders associated with genomic instability, cancer predisposition and features of premature ageing; namely, Bloom's syndrome (*BLM *gene mutations), Werner's syndrome (*WRN *gene mutations), and Rothmund-Thomson syndrome (*RTS*), RAPADILINO and Baller-Gerold syndrome (all caused by mutation of *RECQ4*) [4-8]. Mutations in the *RECQ1 *and *RECQ5 *genes may be responsible for additional cancer predisposition disorders, but this remains to be proven. In this regard, interesting candidates are patients with a phenotype similar to that of *RTS *individuals who do not carry any mutations in the *RECQ4 *gene. Moreover, recent studies have linked a single nucleotide polymorphism present in the *RECQ1 *gene to a reduced survival in pancreatic cancer patients [9,10].

Biochemical studies have demonstrated that RecQ helicases unwind DNA with a 3' to 5' polarity and, although with some differences, are capable of unwinding a variety of DNA structures other than standard B-form DNA duplexes [11,12]. Consistent with an ability to unwind various DNA structures, several cellular functions have been attributed to RecQ proteins, including roles in stabilization and repair of damaged DNA replication forks, telomere maintenance, homologous recombination, and DNA damage checkpoint signaling [13-15].

Previous studies demonstrated that BLM is highly expressed in tumor cells of both lymphoid and epithelial origin and that this reflects the greater fraction of proliferating cells that are present in tumors relative to the normal tissues of the same origin [16]. Similarly, WRN was also recently suggested to be involved in the promotion of tumor cell growth [17]. A cancer specific role of RECQ1 is supported by recent reports showing that RECQ1 silencing in cancer cells resulted in mitotic catastrophe and local and systemic administration of RecQL1-siRNA prevented tumor growth in murine models [18-20].

Here, we characterized the expression of RECQ1 in normal and neoplastic tissues of different origins by immunohistochemical and western blot analysis. Our results show that RECQ1 is highly expressed in tumors. In particular, its expression level increases dramatically in human brain glioblastoma relative to control brain tissues. We also show that RECQ1 depletion affects proliferation of glioblastoma cells and causes an increased level of DNA damages supporting the notion that RECQ1 plays a unique role in the maintenance of genome stability. Moreover, RECQ1 depleted cells are hypersensitive to hydroxyurea (HU) or temozolomide treatment, the latter of which is an anticancer agent widely used for the treatment of human brain tumors. In this regard, glioblastoma is the most common and aggressive histotype of brain tumor with a very poor prognosis [21,22]. One of the reasons why glioblastomas are so difficult to combat is that they are associated with a diffuse invasion of distant tissues by a multitude of migrating gliomas cells characterized by a decreased level of apoptosis and an increased resistance to cytotoxic insults due to the activation of specific signalling pathways [21]. Given that these pathways are not all activated at the same time in all gliomas, the development of specific inhibitors to combat the migratory glioma cells would only be possible if molecular profiling of the tumors of the individual patients is performed. Recent studies reported that the median and 2-year survival rate of patient with glioblastoma was however significantly improved by the addition of concomitant and adjuvant temozolomide to standard postoperative radiotherapy [22]. Temozolomide has the ability to overcome the intrinsic resistance of cancer cells to apoptosis since it works both as a proautoptic and proautophagic cytotoxic drug, thus inducing cell death both by apoptosis and autophagy [23,24]. Moreover, patients characterized by a methylated form of the promoter for the O-6-methylguanine-DNA-methyltransferase (*MGMT*) gene are more likely to benefit from temozolomide treatment [22]. Even if considerable advancements in glioblastoma treatment have been made during the recent years, new therapeutic protocols are highly needed.

## Methods

### Antibodies

The custom rabbit polyclonal anti-RECQ1 antibody against the C-terminal region of RECQ1 (RQ-CT) was raised against a 16 amino acid peptide corresponding to residues 634-649 of RECQ1 (C-SGSKNTGAKKRKIDDA) with an N-terminal cysteine conjugated to the keyhole limpet hemocyanin (KLH) carrier protein (Sigma Genosys). The rabbit polyclonal anti-RECQ1 antibody against the full-length RECQ1 (RQ-FL) was made by injecting the rabbits with full-length recombinant RECQ1 expressed in insect cells [25]. Antibodies against RECQ1 (sc-25547), RAD51 (sc-8349) were from Santa Cruz Biotechnology. Anti-γ-H2AX pSer139 (05-636), anti-BrdU-FITC (347583) and anti-α-Tubulin (T6074) were obtained from Upstate, BD Bioscience and Sigma, respectively. Antibody against Ki-67 (pre-diluted MIB-1, clone 30-9) was from Ventana Medical System. The anti-MGMT (ab39253) antibody was obtained from Abcam. The Glial Fibrillary Acidic Protein (GFAP) antibody (pre-diluted, clone EP672Y) was from Cell Marque. The secondary Alexa 488-, Alexa 594-conjugated antibodies and Toto-3 iodide were from Invitrogen Molecular Probes.

### Cells lines

Human glioblastoma (T98G (ATCC, CRL-1690) and U-87 (ATCC, HTB-14)) and normal human fibroblast (IMR-90) (ATCC, CCL-186)) cell lines were maintained in Dulbecco's modified Eagle's Medium (DMEM) and MEM media supplemented with Glutamax (Life Tecnologies, Inc.) and 10% (v/v) fetal bovine serum (FBS; Life Technologies, Inc.), respectively.

### Tissues

The tumoral and perilesional brain tissues samples were provided by the Neurosurgery Unit of the "Azienda Ospedaliero-Universitaria" of Udine, while the tumoral and perilesional tissues samples of colon, lung, and thyroid plus the normal heart tissue were provided by the ACADEM Department of the Cattinara hospital of Trieste, Italy. The tissues were obtained by surgical resection and they were fixed in formalin and paraffin embedded. In the case of the brain tumors, a portion of the biopsy tissue was kept at -80°C for the subsequent western blot analysis. The tumor diagnosis was confirmed in each case by histopathological analysis. Sections of 4 μm thickness were prepared and mounted on SuperFrost^® ^Plus microscope slides. After overnight drying at 37°C, the slides were processed for immunostaining. The percentage of nuclei showing positive staining with the antibodies was calculated counting three high power fields (HPF) and making the average. The immunostaining with the RQ-FL and RQ-CT antibodies was comparable.

### Immunohistochemistry (IHC)

IHC staining was performed on 4 μm-thick tissue sections that were cut with the microtome from formalin-fixed, paraffin-embedded samples representative of the following tumors: glioblastoma, colon tumors, thyroid tumor and lung tumor. The sections were deposited on SuperFrost^® ^Plus slides and incubated at least for 12 hours at 37°C. The immunostaining procedures were performed automatically using the Benchmark device (Ventana Medical System, Tucson, AZ, USA) and manually, using heat-induced epitope retrieval method. The detection of RECQ1 was performed using the polyclonal antibody produced in rabbit which recognizes the full protein sequence (RQ-FL), or the antibody directed against the last 16 residues at the C-terminus (RQ-CT), both at 1:150 dilution; the immunodetection of Ki-67 was performed using the Ventana pre-diluted MIB-1 antibody and the antibody anti-MGMT was used a 1:150 dilution. All these antibodies were detected using chromogen diaminobenzidine (DAB). For the manual detection, the incubations were performed in a humidified chamber. Briefly, tissue sections, were deparaffinized, immersed in xylene for 30 min, and then hydrated in a decreasing alcohol series. Endogenous peroxidase activity was blocked by incubating the tissue sections in 0.3% H_2_O_2 _for 20 min. For RECQ1 detection, the sections were subject to heat-induced epitope retrieval, immersing the slides in boiling 10 mM citrate buffer, pH 6.0 for 20 min; for Ki-67 staining, we performed heat antigen retrival with 100 mM Tris-Borate, 1 mM EDTA pH 8.0 solution. The sections were incubated for 20 minutes with blocking serum (Vectastain Universal Elite ABC kit, Vector Laboratories, Burlingame, CA, USA) and for 60 minutes with the primary antibody directed against RECQ1, 1:150 diluted. The sections not incubated with the primary antibody were used as negative controls. The slides were washed for some times with PBS and 0.1% of Triton X-100, and incubated for 30 minutes with the biotinylated secondary antibody and with Vectastain ABC system for 30 minutes (Vectastain Universal Elite ABC kit, Vector Laboratories, Burlingame, CA, USA); the detection was made using a solution containing DAB and H_2_O_2 _(DAB substrate kit, Vector Laboratories, Burlingame, CA, USA). The sections were counterstained with Mayer hematoxylin. Preadsorption of the antibody, using a molar excess of the specific immunizing peptide (1:5 at 37°C for 30 min) was performed to verify the antibody specificity.

### Glioblastoma tissue microarray construction

Multiform glioblastoma (GBM) tissue samples were collected from the archives of the Pathology Department of the Cattinara Hospital of Trieste, Italy. A tissue microarray (TMA) was constructed collecting 63 lesional and 19 perilesional tissues; specifically, the array had a total of 82 cores. The tissue cores were related to 63 patients whose diagnosis was primary glioblastoma. These patients were randomly selected from the clinical records of the Cattinara Hospital from 01/01/2003 to 31/12/2009. The selection criteria were a primary diagnosis of glioblastoma, no relapses or metastases were inserted in the TMA. Among the 63 patients, 29 were males with a median age of 65 years (range 42-84) and 33 were females with a median age of 62 years (range 42-81).

Areas showing the histopathologic features of GBM and perilesional tissue were selected on archival hematoxylin and eosin (H&E) slides, and marked for TMA construction. Briefly, after the tissue cylinders of 1.5 mm in diameter were taken from the selected regions of the donor paraffin block, they were punched into a recipient paraffin block using a tissue-arraying instrument (Galileo TMA CK3500, from by Integrated Systems Engineering, Milano, Italy). Sections of 4 μm thick were prepared and mounted on microscope slides. After overnight drying at 37°C, the slides were processed for immunostaining. An H&E stained section was also made to confirm the presence of the original areas selected from each tumor. The TMA sections were evaluated by two pathologists to validate the diagnostic morphology of each array spot.

The TMA sections were stained with RECQ1 (diluted 1:150, RQ-CT), Ki-67, MGMT and the Glial Fibrillary Acidic Protein (GFAP) antibodies for IHC analysis. The antibodies were detected using chromogen diaminobenzidine (DAB). The procedure for IHC analysis of the TMA was the same described in the paragraph above with the exception that a buffer containing 100 mM Tris-Borate, 1 mM EDTA pH 8.0 was used for the antigen retrieval in the case of Ki-67 and GFAP. IHC against GFAP, which is a specific marker of the astrocytes, allowed discriminating the astrocytes from oligodendrocytes and support neurons (negative to GFAP staining). During the cell counting for RECQ1, only the astrocytes' positivity was considered. The percentage of nuclei showing positive staining with the antibodies was calculated counting three high power fields (HPF) and making the average.

### Analysis of the genetic status of *MGMT *and *IDH *genes

For the qualitative detection of *MGMT *gene promoter methylation status, DNA was extracted from FFPE tissues of each tumor samples. The tissues' specimens were deparaffinized, DNA was extracted and chemically modified by bisulfite treatment for subsequent methylation specific PCR analysis according to a previously described protocol [26]. For sequence analysis of typical somatic mutations in the *IDH1 *and *IDH2 *genes, DNA from tissues' specimens was extracted following the same protocol used for DNA methylation analysis, while for the T98G and U87 cell lines DNA was extracted according to the manufacture's instructions using the Wizard Genomic DNA purification Kit (Promega). PCR analysis was performed as already reported [27]. Amplicons were purified by a PCR purification kit (Qiagen) and sequenced by Sanger's method.

### Statistical Analysis

The Wilcoxon test (non parametrical) for matched-samples was used to evaluate differences in IHC analyses between tumor tissues and corresponding normal peri-lesional tissues. IHC results considered in this analysis were: the number of positive cells for RECQ1, the intensity of the signal and the number of positive cells for Ki-67. Spearman's correlation test was applied to assess the relationship between intensity score of RECQ1 and the number of positive cells in lesional tissues. Kruskal-Wallis test was applied to investigate differences in the percentage of positive cells for Ki-67 and RECQ1 among groups defined by the gender of the patients and the intensity score of RECQ1. The above statistical tests were also applied to investigate if there was any correlation between the percentage of RECQ1 positive cells and the methylation status of the *MGMT *gene or the expression level of the MGMT protein in primary glioblastomas showing an intensity score of 3+ for RECQ1. Two-sided probability values of less than 0.05 were considered to indicate statistical significance. Statistical analyses were performed using Stata SE 9.2 software (Stata Corp College Station, TX, USA).

### Cells and cell cycle analysis

Glioblastoma (T98G and U-87) and normal human fibroblast (IMR-90) cell lines were maintained in DMEM and MEM media supplemented with Glutamax (Life Tecnologies, Inc.) and 10% (v/v) fetal bovine serum (FBS; Life Technologies, Inc.), respectively. BrdU incorporation experiments were performed on transiently siRNA transfected cells 72 h post-transfection. Cells were pulsed for 1 h with BrdU (Sigma, final concentration 10 μM), and BrdU-positive cells were detected by using a mouse anti-BrdU-FITC primary antibody followed by an anti-mouse Alexa 488-conjugated secondary antibody. Cells were collected and analyzed by flow cytometry on a FACSCalibur (Becton Dickinson) to simultaneously determine the cell-cycle profile (DNA content) by incorporation of propidium iodide (Sigma) and the S-phase cell population by incorporation of BrdU. Cell cycle profile distributions were determined with the CellQuestPro and Modfit LT 3.0 software.

### Immunoblotting

Brain tissues extracts were prepared in buffer TNEN (50 mM Tris pH 7.5, 150 mM NaCl, 2 mM EDTA, 0.2% triton X-100 and 0.3% NP-40) following a previously described procedure [28]. T98G, U-87, and IMR-90 whole cell extracts were prepared in HNNG buffer (15 mM Hepes pH 7.5, 250 mM NaCl, 1% (v/v) NP-40, 10% (v/v) glycerol, 1 mM PMSF) supplemented with 0.2 mM sodium orthovandate (Sigma), 10 mM sodium glycerol-2-phosphate (Sigma), 25 mM NaF (Sigma) and protease inhibitors cocktail tablet (Roche). Immunoblots were carried out with 2 to 10 μg of whole-cell lysates. Proteins were separated on 12% SDS-PAGE gel and detected by immunoblotting using the SuperSignal West Femto Maximum Sensitivity Substrate (Pierce).

### RNA interference

Cells were transiently transfected with a commercial mix of 4 siRNAs against RECQ1 (NM_032941: Dharmacon-SMARTpool; target sequences: GAGCUUAUGUUACCAGUUA, CUACGGCUUUGGAGAUAUA, GAUUAUAAGGCACUUGGUA, GGGCAAGCAAUGAAUAUGA) for 72 hours at 100 nM final concentration using the Hyperfect transfection system (QIAGEN) and following the manufacturer's instructions. The specificity of this siRNA mix was already confirmed in our previous study [29]. RNAi control experiments were performed using a siRNA against Luciferase (Dharmacon). Alternatively, an efficient reduction of the expression level of RECQ1 in the T98G cells was also obtained by transfection with a pcDNASup plasmid (hybrid plasmid obtained by combination of pcDNA3) (Invitrogen, life technologies) and pSUPER (OligoEngine) encoding a shRNA (short hairpin RNA) against RECQ1 mRNA (target sequence (target): 5'-GAGCUUAUGUUACCAGUUA-3'). The T98G cell clones downregulated for RECQ1 were obtained by selection with Geneticin G418 antibiotic (0.5 mg/L) (GIBCO BRL).

### Clonogenic assays

Colony forming assays were conducted in vitro as already described [30]. Briefly, the assays were performed in six-well plates, with clones produced either by transfection with a luciferase-siRNA duplex as control or with a RECQ1-siRNA. Additionally, clonogenic assays were carried out on stable clones produced by T98G cells transfection with empty pcDNASup vector or clones obtained from T98G cells transfected with the pcDNASup containing RECQ1-shRNA. Cells were seeded at different dilutions (100, 200, 400, 800, 1600 and 3200 cells per well) in six-well plates, and the colonies formed after at least one week of growth were counted using VersaDoc 4000 imaging system (BioRad). Proliferative capacity of control cells and of the RECQ1-depleted cells was evaluated by their plating efficiency (PE) and expressed as colony forming capacity. The PE was calculated as the average ratio of the number of formed colonies *versus *the number of cells seeded, expressed as percentage. Cell survival assays were performed by plating transfected cells before treatment with hydroxyurea (HU: Sigma) or temozolomide (TMZ: Sigma). Cells were treated overnight with different doses of HU (0.2, 2, 10 and 20 mM) or TMZ (5, 50, 250 and 500 μM). Surviving fractions were calculated following a procedure already published [30].

### Immunofluorescence

Briefly, cells were seeded in chamber slides (NALGENE) after transient downregulation of the level of RECQ1 expression by RNA interference for 24 hours. A second transfection with the siRNA was repeated immediately after the cells were seeded in the chamber slides. The cells were left under these conditions for 72 hours. The medium was then removed and the chamber slides were washed, fixed and immunostained by a set of specific antibodies following a protocol already described [31]. The primary antibodies used in this study were already described above. The primary antibodies used for the immunofluorescence analysis are described in the first "Material and Methods" paragraph. The slides were then incubated with the secondary Alexa 488 and Alexa 594 fluorophores-conjugated antibodies (Invitrogen, Molecular Probes). Confocal fluorescence analysis was performed on a Zeiss LSM 510 Meta confocal microscope. Images were acquired using the LSM software. The cells containing γ-H2AX and RAD51 foci were counted in at least 100 nuclei.

## Results

### Expression of RECQ1 in human tumor tissues

**Figure 1:**
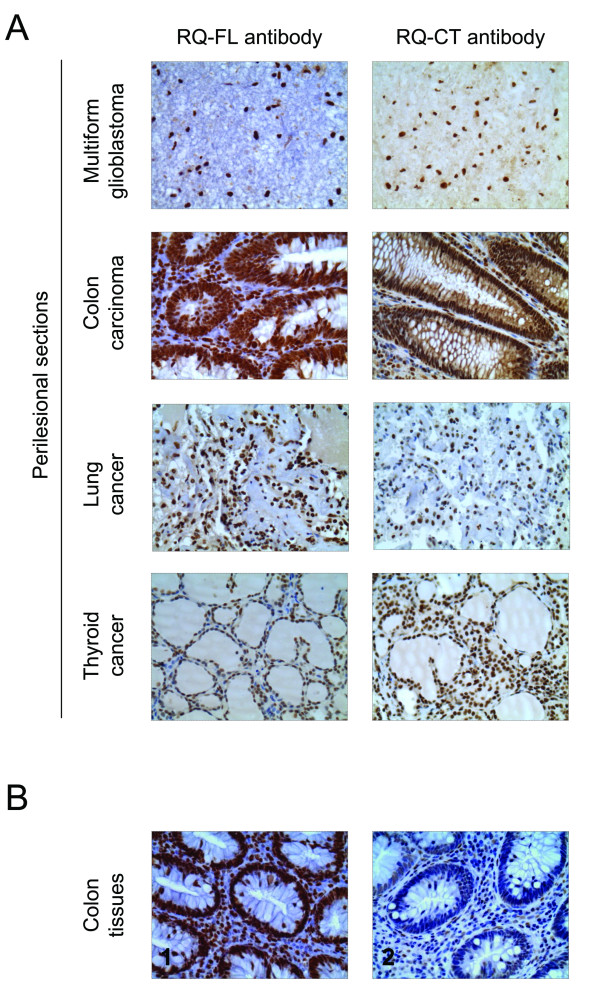
**Comparison of the efficiency of the RQ-FL and RQ-CT antibodies**. A) Representative immunostaining against the RQ-FL antibody (left) and the RQ-CT antibody (right). All tissues are perilesional sections from multiform glioblastoma, colon carcinoma, lung cancer and thyroid cancer. The nuclei positive for the antibody are in brown and the tissues were counterstained with hematoxylin (O.M 40X). B) Pre-adsorption experiments using the recombinant RECQ1 protein on normal colon tissues. Immunostaining against RQ-FL antibody in colon tissues before (1) and after preadsorption with the full-length recombinant RECQ1 protein (2). The nuclei positive for the antibody are in brown and the tissues were counterstained with hematoxylin (O.M. 40X).

**Figure 2:**
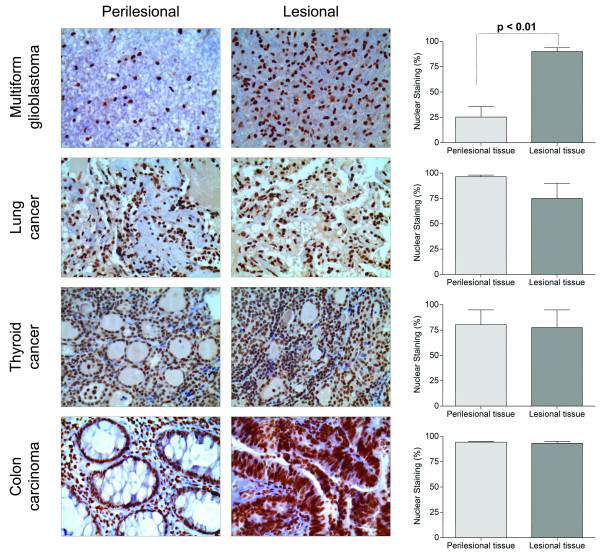
**RECQ1 expression in tumors of different origins**. A) Representative micrographies of immunohistochemical analysis on tissues from different types of tumors. On the left, the perilesional zone and, on the right, the tumoral one. Immunostaining was made using the anti-RECQ1 RQ-FL antibody. The nuclei positive for the protein are in brown and the tissues were counterstained with hematoxylin (O.M 40X). The histograms on the right represent the percentage of nuclei positive for RECQ1 in perilesional and lesional tissues of the indicated tumors.

Immunohistochemical analysis of perilesional sections of human colon carcinoma, thyroid cancer, lung cancer, and brain glioblastoma tissues showed that RECQ1 was effectively detected in these samples. The RECQ1 expression was confined in the cellular nuclei and more than 30% of cells in each sample stained positive for RECQ1 using either an antibody raised against the full-length protein (RQ-FL) or an antibody specifically recognizing the C-terminus of RECQ1 (RQ-CT) (Figure 1A). The specificity of the two anti-RECQ1 antibodies was confirmed by pre-adsorption experiments showing that after pre-adsorption of the tissue with the recombinant RECQ1 protein only a minimal non-specific stain could be detected (Figure 1B). The immunohistochemical analysis was then repeated including lesional tissues from brain glioblastoma, colon carcinoma, lung and thyroid cancers for comparative analysis (Figure 2). The results showed that RECQ1 was highly expressed both in the perilesional sections and in lesional sections of these tumors, with the notable exception of brain gliomas where the expression of RECQ1 was significantly higher in the tumor samples relative to the perilesional tissues. In particular, we observed a 4-fold decrease in the percentage of RECQ1 positive nuclear staining cells from brain tumor sections *versus *perilesional tissue sections.

**Figure 3:**
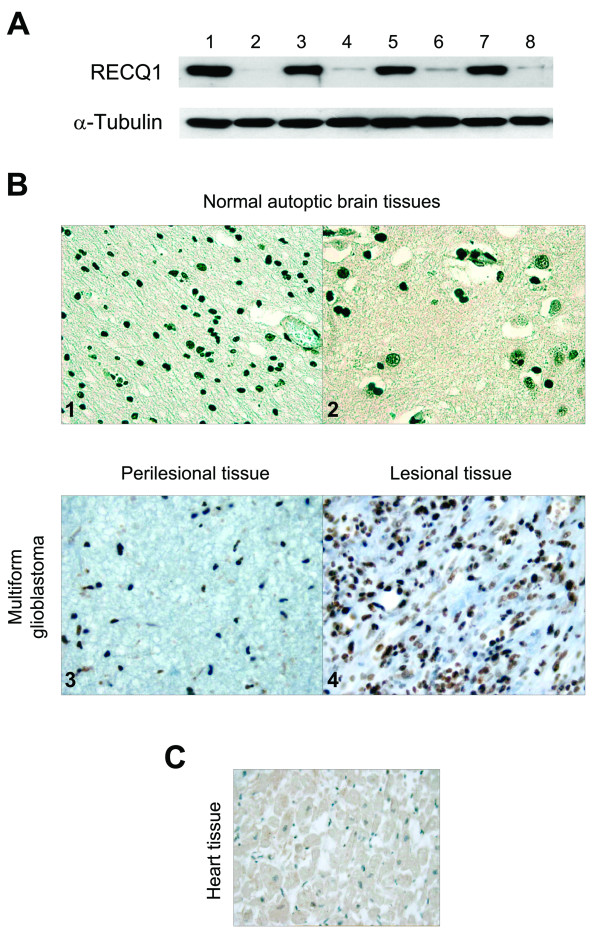
**RECQ1 expression in human brain glioblastomas**. A) Western blot analyses of surgically removed perilesional (lanes 2,4,6,8) and lesional tissues from glioblastoma multiforme (lanes 1,3,5,7) performed using the anti-RECQ1 RQ-FL antibody. Alpha-tubulin protein was used as an internal control to guarantee that the amount of proteins loaded on each well was the same. B) Representative immunostaining of RECQ1 expression in normal autoptic brain tissues (top), and perilesional and lesional glioblastoma tissues (bottom) performed using the anti-RECQ1 RQ-FL antibody and the Benchmark automatic immunostaining device (Ventana Medical System, Tucson, AZ, USA). Immunostaining was made using the anti-RECQ1 RQ-FL antibody. The nuclei positive for RECQ1 are in brown and the tissues were counterstained with hematoxylin (normal autoptic brain tissues, O.M 40X; perilesional and lesional glioblastoma tissues, O.M. 40X). B) Immunostaining for RECQ1 on human normal heart tissue counterstained with hematoxylin (O.M 20X).

The higher expression of RECQ1 in brain glioblastoma was confirmed by Western blot analysis showing that RECQ1 was at least 10-fold more abundant in the tumoral tissues compared to the perilesional samples, where RECQ1 was almost undetectable (Figure 3A). The same results were also confirmed using autoptic normal brain tissues, which might represent a more appropriate control for our experiments since we cannot rule out the possibility that the perilesional tissues might be partially infiltrated with the tumor. The fact that the perilesional and autoptic normal brain tissues showed a lower number of positive nuclei compared to the control tissues from other types of tumors suggests that RECQ1 is less expressed in normal non-dividing tissues (Figure 3B). This hypothesis was confirmed by the analysis of a human heart tissue, which is another classical example of a non dividing tissue, and did not show any significant nuclear staining for RECQ1 (Figure 3C). Collectively, our immunohistochemical analysis of different tumor samples indicates that RECQ1 is highly expressed in all types of tumors. However, only in the case of glioblastoma this high expression parallels with its lower expression in the perilesional counterpart due to the extremely low expression of RECQ1 in non-dividing tissues.

### Tissue microarray analysis

**Figure 4:**
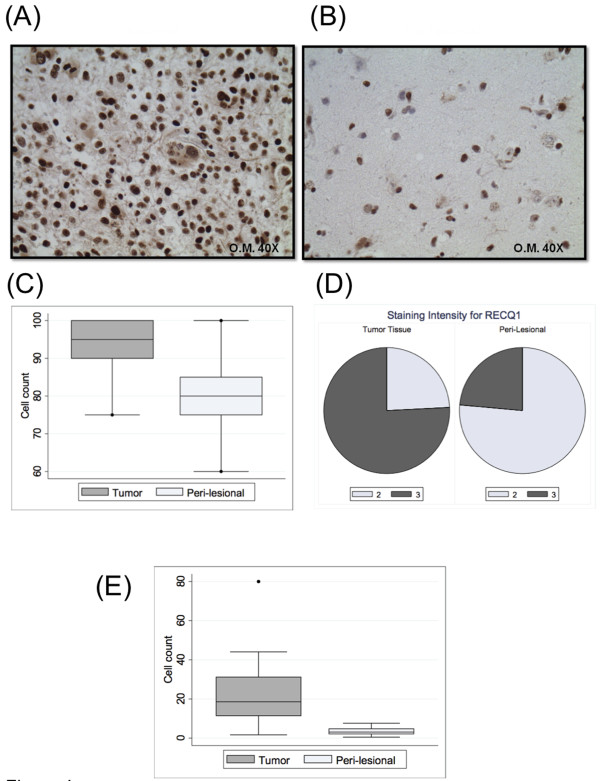
**RECQ1 expression in TMA from human brain glioblastomas**. A) Representative immunostaining of RECQ1 expression in tissue core derived from a glioblastoma specimen and B) corresponding normal surrounding tissue (magnification 40×). C) Box plot representing the count of cell positive for RECQ1 in 19 brain glioblastomas and paired peri-lesional tissues independently of the staining intensity of the samples. D) Pie chart presenting the distribution of the staining intensity for RECQ1 in 63 brain glioblastomas and 19 paired peri-lesional tissues E) Box plot representing the count of cell positive for Ki-67 in 19 brain glioblastomas and paired peri-lesional tissues. The boxes summarize the confidence interval (lower and higher horizontal lines), 25^th^-50^th ^and 75^th ^percentiles (horizontal lines of the box).

To validate our conclusion that RECQ1 is highly expressed in glioblastomas, we analyzed the expression pattern of RECQ1 on a tissue microarray containing a total of 63 primary glioblastoma and 19 perilesional tissues (Figure 4A,B). In agreement with the previous results, the percentage of RECQ1 positive cells (95%) and the intensity of the staining was significantly higher in tumoral *versus *perilesional tissues (P = 0.0013 and P = 0.0009, respectively) (Figure 4C,D). The higher positivity of primary glioblastoma tissues for RECQ1 correlated with higher intensity of the immunostaining. In particular, the RECQ1 positive cells of 48 out of 63 glioblastoma speciemen present in the TMA showed a strong nuclear positivity characterized by a staining nuclear intensity of 3+, while most of the cells positive for RECQ1 in the perilesional tissues were characterized by a weaker staining intensity of 2+ (Figure 4D). As expected, the number of positive cells for Ki-67 differed significantly between tumors and matched surrounding normal tissue, with an higher expression of the protein in the lesional tissues (P = 0.0008) (Figure 4E). GFAP staining confirmed that in the perilesional area the majority of cell positive to RECQ1 were astrocytes, even though some positive staining for RECQ1 was detected also in the oligodendrocytes and the support neurons (Additional File 1). However, GFAP staining demonstrated that only astrocytes presented an intense nuclear positivity to RECQ1 in tumor tissues.

A detailed IHC analysis of the glioblastoma tissues present in the TMA indicated that the percentage of positive cells for Ki-67 and RECQ1 did not differ significantly between men and women (P = 0.9 and 0.7, respectively). Similarly, the age at diagnosis did not correlate with the percentages of positive cells for RECQ1 and Ki-67 (P = 0.4 and P = 1.0), nor with the intensity of the signal for RECQ1 (P = 0.5). A significant trend was detected between the percentage of positive cells and the intensity of the signal for RECQ1 (Spearman's rho = 0.5, P = 0.0001) indicating that a high number of positive cells is associated with a stronger signal. No correlation was present between the percentage of positive cells for RECQ1 and Ki-67 (Spearman's rho = 0.2, P = 0.2).

Previous studies indicated that the methylation of the *MGMT *gene promoter is a strong predictor of the benefit from temolozolomide chemotherapy [22]. In order to test if there was any relationship between RECQ1 expression and *MGMT *status, we investigated the methylation status of the *MGMT *gene promoter region in the subgroup of RECQ1 positive primary glioblastomas characterized by a staining nuclear intensity value in IHC of 3+. Statistical analysis on this set of data clearly showed that there was no correlation between the percentage of RECQ1 positive cells and the methylation status of the *MGMT *promoter region in primary glioblastoma tissues (P = 0.3757). Similar results in terms of no statistical significance were obtained when considering the status of MGMT protein expression in the same subgroup of glioblastomas (P = 0.2309) (Additional File 2). Recent mutational analyses also revealed that somatic mutations in the NADP-dependent isocitrate dehydrogenase genes, *IDH1 *and *IDH2*, were associated with an increased overall survival of glioblastoma patients [27,32,33]. However, these mutations are rare in primary glioblastomas (grade IV)--the group of gliomas used in this work-- while are most frequent in tumors that evolved from lower-grade gliomas (secondary glioblastomas) [27,33]. In agreement with these findings, our preliminary analysis of the *IDH *genes showed that both *IDH1 *and *IDH2 *genes were wild type in our group of primary glioblastoma tissues (data not shown). In summary, our TMA analysis confirmed that the expression of RECQ1 is significantly increased in the glioblastoma tissues relative to perilesional tissues. Moreover, RECQ1 protein overexpression does not seem to have a prognostic value associated with *MGMT *methylation status in primary glioblastoma multiforme.

### RECQ1 silencing inhibits cell proliferation in glioblastoma cells

**Figure 5:**
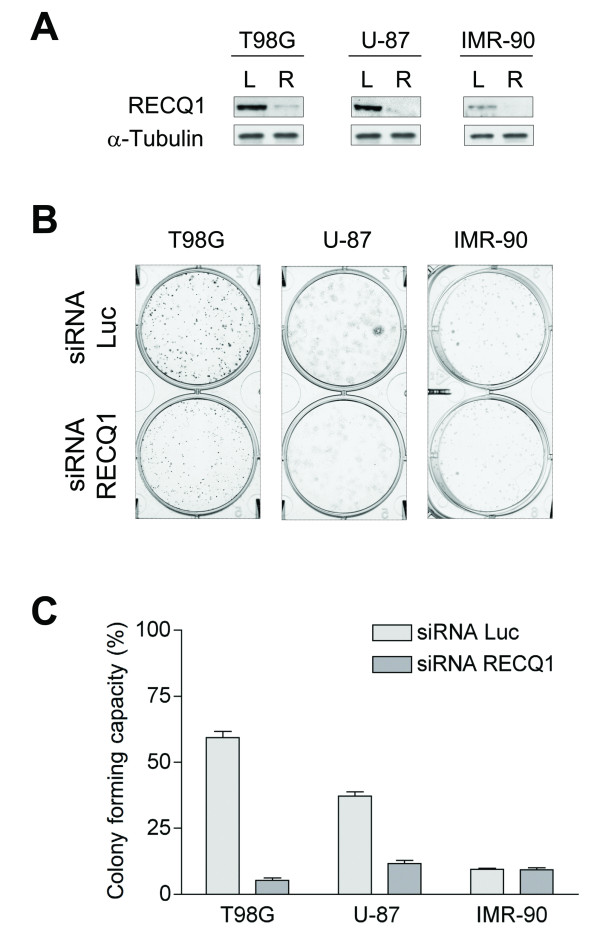
**Proliferative capacity of RECQ1-depleted glioblastoma cells**. A) Western blot analysis of T98G, U-87 and IMR-90 cell lines transiently transfected with an siRNA against RECQ1. L indicates the Luciferase siRNA control, R the siRNA against RECQ1. B) Clonogenic assays performed in RECQ1-depleted T98G, U-87, and IMR-90 cell lines. Pictures show colonies formed after seeding 800 cells. C) Graphs showing the plating efficiencies expressed as colony forming capacity. Values represent the average ratio of the number of formed colonies to the number of cells seeded, expressed as percentage.

To test the role of RECQ1 in glioblastoma cell growth and proliferation, we compared the colony forming properties of two different glioblastoma cell lines (T98G and U-87), and normal human IMR-90 fibroblasts, transiently transfected with RECQ1-specific siRNAs *versus *cells transfected with a luciferase siRNA duplex, as control (Figure 5). More than 80% depletion of RECQ1 was observed in all whole cell extracts upon treatment with the RECQ1-specific siRNA duplex as compared to the control cells (Figure 5A). Our colony forming assays demonstrated a significant reduction in both the size and number of colonies of RECQ1 downregulated T98G and U-87 glioblastoma cells (Figure 5B,C). However, the downregulation of RECQ1 in normal human primary fibroblasts did not significantly affect the proliferation capacity of these cells in agreement with previous findings [19].

**Figure 6:**
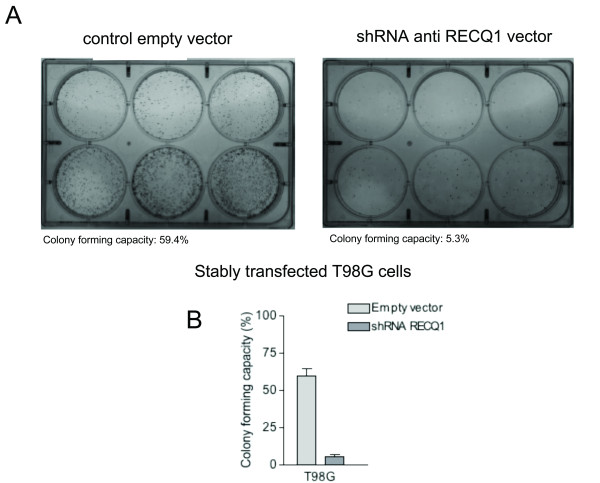
**Proliferative capacity of T98G cells stably transfected with a shRNA against RECQ1**. A) Clonogenic assay performed in either control cells (left plate) and in clones silenced for RECQ1 (right plate). The figure shows colonies formed after plating 100, 200, 400, 800, 1600 and 3200 cells (well 1 to well 6, respectively). B) Graph showing the plating efficiencies expressed as colony forming capacity. Values represent the average ratio of the number of formed colonies to the number of cells seeded, expressed as percentage.

The same experiments were then repeated on a T98G cell line stably transfected with a plasmid codifying a RECQ1-specific shRNA (Figure 6). A comparison of selected G418 resistant clones that carry the plasmid codifying the RECQ1 shRNA with control cells containing the empty vector enabled us to assess the effect of stable RECQ1 depletion on the colony forming ability of RECQ1-depleted T98G cells. In agreement with the results obtained with the siRNA mix, our colony forming assays showed a significant reduction in both size and number of colonies when T98G cells were stably downregulated for RECQ1. In particular, RECQ1-depleted glioblastoma cells showed ~10 fold reduction in their proliferative capacity in comparison with control cells (from 5.3% to 59.4%). Collectively, our data point to a specific regulatory role of human RECQ1 in the proliferation in glioblastoma cells.

### RECQ1 depletion leads to cell cycle perturbation in T98G glioblastoma cells

**Figure 7:**
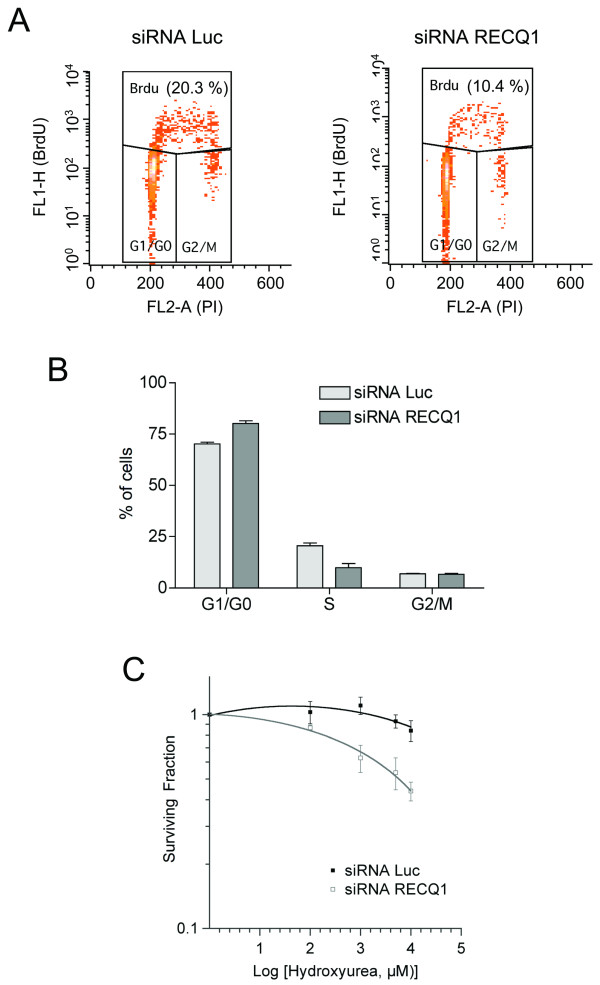
**Downregualtion of RECQ1 leads to defects in cell cycle progression and DNA synthesis in glioblastoma T98G cells**. A) Flow cytometry profiles of DNA content (x-axis; propidium iodide/PI staining) versus BrdU incorporation (y-axis; anti-BrdU immunostaining) 72 h after siRNA transfection. Boxes are labeled to indicate cell cycle phases. B) The bar graph at bottom reports the percentage of G0/G1, S-phase/BrdU positive and G2/M cells in cultures that had been transfected with RECQ1 or luciferase/control siRNA pools. Results shown are the mean ± SEM from three independent experiments. C) The graph shows the cellular surviving fractions measured at different doses of hydroxyurea in control and RECQ1-depleted T98G cells. Surviving fraction values are the mean ± SEM from three independent experiments.

Previous studies showed that siRNA-mediated depletion of RECQ1 impairs cellular proliferation in different cell lines [19]. This notion was also supported by our FACS analysis of RECQ1-depleted T98G glioblastoma cells that had been bromodeoxyuridine (BrdU)-labelled, indicating that there is more than 50% reduction in both BrdU labelling and S phase fraction. This reduction of S phase cells is associated to an increased fraction of cells arrested in G1 (Figure 7A and 7B). These results confirm that RECQ1 depletion suppresses cell proliferation, and may do so by interfering with DNA synthesis.

In order to provide additional data on the role of RECQ1 in DNA synthesis and cell proliferation, we analysed the colony forming capacity of RECQ1-depleted cells upon replication stress induction with hydroxyurea. Cellular survival curves at increasing hydroxyurea concentrations showed that RECQ1 depleted cells were hyper-sensitive to hydroxyurea treatment suggesting a possible role of RECQ1 in DNA replication fork processing (Figure 7C).

### RECQ1 depletion in glioblastoma cells results in an increased load of DNA damages

**Figure 8:**
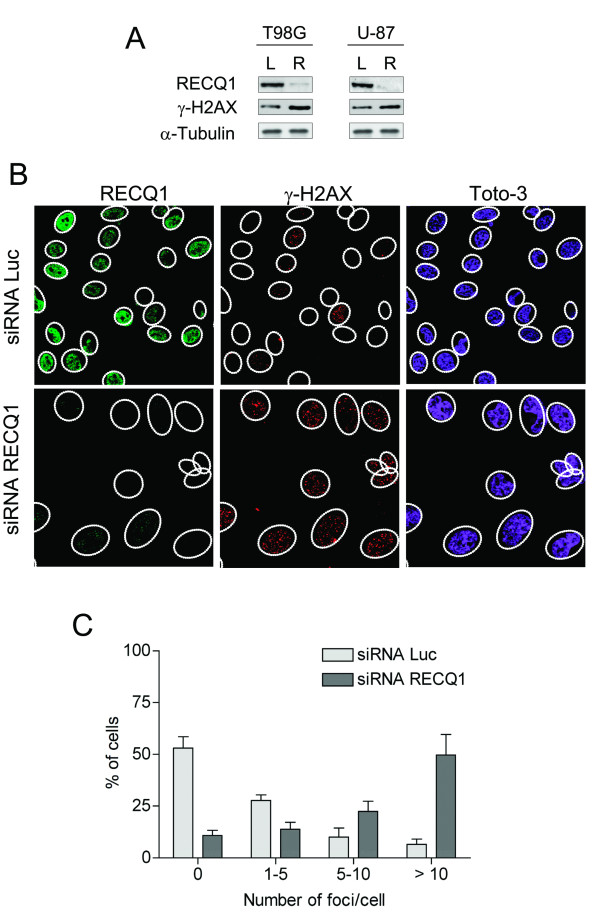
**Downregulation of RECQ1 triggers spontaneous γ-H2AX foci accumulation**. A) Western blot analysis of T98G cell lines transiently transfected with an siRNA against RECQ1. L indicates the Luciferase siRNA control, R the siRNA against RECQ1. B) Immunostaining of endogenous RECQ1 and endogenous γ-H2AX on T98G cells after treatment with anti-RECQ1 siRNA or control siRNAs. C) The graph shows the percentage of cells that contain a defined number of γ-H2AX foci per cell.

**Figure 9:**
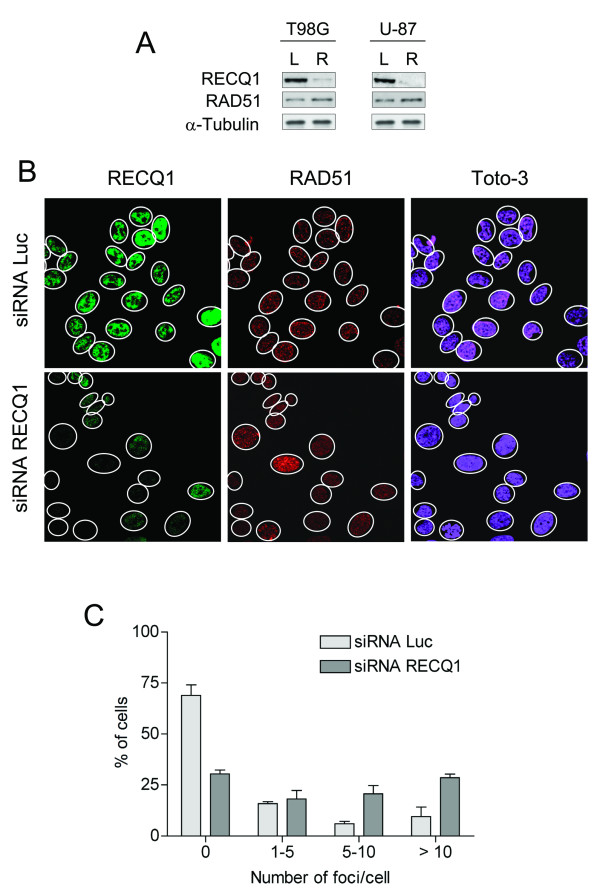
**Downregualtion of RECQ1 triggers spontaneous RAD51 foci accumulation**. A) Western blot analysis of T98G cell lines transiently transfected with a RECQ1-specific siRNA duplex. L indicates the Luciferase siRNA control, R the siRNA against RECQ1. B) The pictures show the immunostaining of endogenous RECQ1 and RAD51 on T98G cells after treatment with anti-RECQ1 siRNA or control siRNAs. C) The graph shows the percentage of cells that contain a defined number of RAD51 foci per cell.

RecQ helicases are involved in the stabilization and repair of damaged DNA replication forks in response to endogenous or exogenous stress [34]. A failure to stabilize forks can lead to fork collapse and DNA breaks [35]. Consistently, previous studies with RECQ1 deficient HeLa cells showed an increased level of DNA damage and sister chromatid exchanges upon RECQ1 depletion [36]. To confirm that RECQ1 has the same role in glioblastoma cells, we analyzed the extent of spontaneous γ-H2AX foci formation in control or RECQ1 siRNA transfected T98G and U-87 cells (Figure 8). The phosphorylated form of histone H2AX (γ-H2AX) is a well-known marker of DNA breaks in cells [37]. Our immunoblots using an antibody against γ-H2AX confirmed previous observations of an increased load of DNA lesions upon RECQ1 depletion (Figure 8A). Consistently, our immunofluorescence experiments indicated that RECQ1 depletion results in a dramatic increase in spontaneous γ-H2AX foci formation confirming that the reduced expression of RECQ1 is associated with defects in DNA repair (Figure 8B,C). Approximately 50% of the RECQ1 depleted cells contained more than ten γ-H2AX foci per nucleus, compared to only 15% of the wild-type cells. Previous studies suggested that RecQ helicases play an important role in homologous recombination (HR) repair at sites of chromosomal DNA damage [3]. To test if RECQ1 is also involved in this pathway, we analyzed the ability of RAD51, a protein involved in the strand invasion step of HR repair, to form foci in RECQ1 depleted T98G cells. As shown in Figure 9, the RECQ1 depleted cells exhibited an increased number of spontaneous RAD51 foci relative to the control cells. However, the increase in the number of RAD51 foci is less pronounced compared with the γ-H2AX foci suggesting the double-strand breaks might not be the major form of damage that leads to γ-H2AX foci formation in the absence of RECQ1.

### RECQ1 depleted glioma cells are hypersensitive to temozolomide treatment

**Figure 10:**
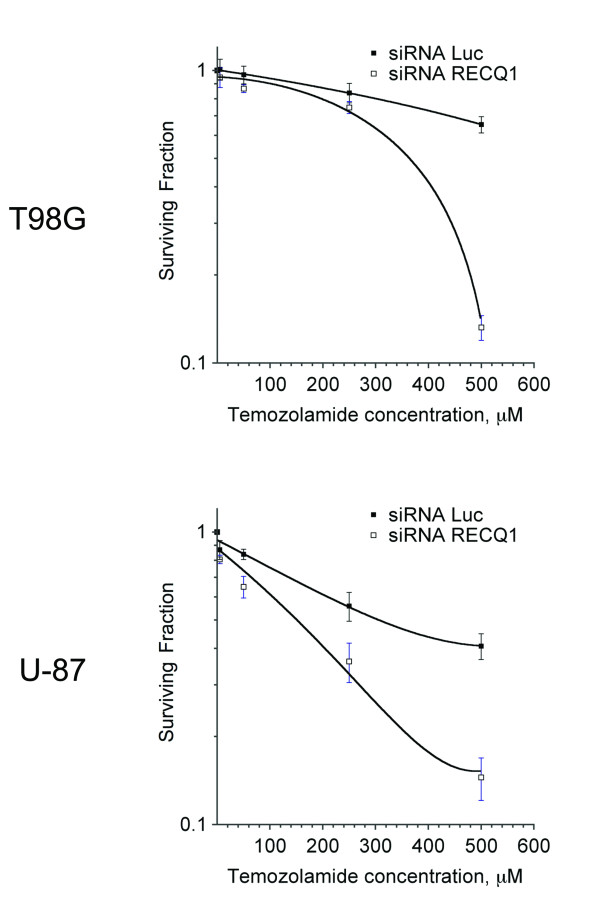
**RECQ1 downregulated glioblastoma cells are hypersensitive to TMZ treatment**. The graphs show the cellular surviving fractions measured at different doses of temozolomide in control and RECQ1-depleted T98G and U-87 cells. Surviving fraction values are the mean ± SEM from three independent experiments.

To explore the possibility that RECQ1 might represent a suitable new target for brain tumor treatment, we investigated the sensitivity of glioblastoma cells to temozolomide (TMZ), which is a commonly used anticancer agent for the treatment of human brain tumors [21-23]. TMZ is an alkylating agent that effectively inhibits glioblastoma cell proliferation. Its toxicity is primarily due to formation of O6-methylguanine in DNA, which mispairs with thymine during DNA replication cycles and after accumulation of unrepaired DNA mismatches results in cell death [38,39]. Thus, we analysed the colony forming capacity of RECQ1-depleted glioblastoma cell lines after treatment with TMZ (Figure 10). Cellular survival curves using increasing TMZ concentrations showed that RECQ1 depleted T98G and U87 cell lines were hypersensitive to the action of TMZ suggesting a possible role of RECQ1 in DNA repair pathways linked to DNA replication. These data also suggest that RECQ1 might represent a suitable new target for the inhibition of cell proliferation in brain tumors.

Given that the status of *MGMT *and *IDH *genes are central in the treatment and/or prognosis of glioblastoma tumors [32], we investigated the methylation status of the *MGMT *gene and the presence of stereotypical mutations of *IDH *genes in the T98G, U-87 and IMR90 cell lines that were used in the RNA interference experiments (Additional File 3). Our results showed that the T98G and U-87 glioblastoma cell lines were both methylated at the *MGMT *gene promoter, while the primary human fibroblasts IMR-90 had unmodified *MGMT *alleles (Additional File 3A). These results indicate that the T98G and U-87 cells are likely to be more susceptible to the cytotoxic effect of temozolomide treatment compared to cells carrying unmodified *MGMT*. On the other hand, sequence analysis showed that the residues R132 and R172 of the *IDH1 *and *IDH2 *genes, respectively, were no mutated in all cell lines analyzed (Additional File 3B). Moreover, the *MGMT *promoter methylation status and stereotypical mutated codons in *IDH *genes did not change upon RECQ1 silencing after RNA interference indicating that the changes in TMZ sensitivity observed upon RECQ1 downregulation are not associated with modifications in the *MGMT *and *IDH *genes.

## Discussion

In this work, we used immunohistochemical and western analysis to show that RECQ1 is highly expressed in human brain glioblastoma and it is confined in the nuclei of the tumor cells, suggesting that this protein plays an important role in glioblastoma tumor growth. This conclusion was validated on a tissue microarray containing a total of primary 63 glioblastoma and 19 perilesional tissues and indicated that the higher expression of RECQ1 in the tumors is not related to the sex or the age of the individuals. RECQ1 expression is also not related to the methylation status of the *MGMT *gene which identifies a subgroup of glioblastoma more susceptible to the cytotoxic effect of temozolomide treatment. Interestingly, we see that RECQ1 is also highly expressed in other types of tumors, in agreement with previous findings [40,41]. However, only in the case of brain tumors, the high expression of RECQ1 is paralleled to a reduced expression of the same protein in perilesional and/or normal tissues, possibly due to the low degree of proliferation of brain cells. Hence, we believe that RECQ1 would be an ideal target for chemotherapy especially in the case of brain tumors since its depletion by RNAi, or its inhibition by selective compounds, would primarily affect tumoral cells.

An essential role of RECQ1 in tumor growth and proliferation is confirmed by our clonogenic assays where we noticed a significant reduction in the number of colonies when T98G and U87 cells were inhibited for the expression of RECQ1. This notion is supported by our previous observation that RECQ1 plays an important role during DNA replication, which is distinct from that of other human RecQ helicases [29]. In addition, we previously showed that RECQ1, BLM, and WRN are characterized by different substrate specificities [42]. In agreement with these findings, Sharma et al. showed that RECQ1 is important for HeLa cell proliferation and plays a unique role in the maintenance of genome integrity [36,43]. Consistently, we find that the depletion of RECQ1 results in spontaneous γ-H2AX foci formation and HU hypersensitivity in T98G cells, suggesting that RECQ1 plays an important and unique role in DNA repair during DNA replication. Concerning the specific role of RECQ1 in genome maintenance, Sharma et al. suggested that RECQ1 might be involved in the regulation of the homologous recombination pathway of DNA double-strand break repair [36]. Interestingly, our immunofluorescence experiments indicate that RECQ1 loss results in fewer RAD51 foci compared to γ-H2AX foci indicating that RECQ1 might not play a major role in homologous recombination (HR). Consistently, we do not observe any significant defect in HR frequency in RECQ1 siRNA-inhibited cells (indeed, HR frequency was slightly increased upon RECQ1 depletion) (data not shown). The hyper-recombination phenotype of RECQ1-depleted cells suggests either that RECQ1 is involved in the suppression of some illegitimate recombination events, as already proposed for other helicases of the same family [44], or that loss of RECQ1 results in the accumulation of some form of DNA lesion or strand breaks, other than DSBs, that might subsequently lead to repair by HR if not properly repaired.

The resistance of glioma cells to TMZ is mainly associated with levels of the DNA repair protein O^6^-alkylguanine alkyltransferase (AGT), which removes alkyl groups at the O^6 ^position of guanine. In fact, O6-benzylguanine (O6-BG), an inhibitor for AGT, reduces resistance to TMZ [45]. Moreover, it has been demonstrated that chemosensitivity of tumor cells to TMZ correlates with the inhibition of telomerase activity [46]. Our studies using malignant glioma cell lines with low (U87-MG) and high levels of AGT (T98G), show that RECQ1 suppression by RNA interference increases the sensitivity of these cells to TMZ, independently of the AGT expression levels. Our data show also that the hypersensitivity of the RECQ1 depleted cells to TMZ is not linked to the methylation status of the *MGMT *gene which is methylated in both T98G and U87 cells or to the presence of stereotypical mutations in the *IDH1 *and *IDH2 *genes which are absent in these cells. The increased sensitivity of RECQ1 depleted glioblastoma cells to TMZ support the notion that RECQ1 plays a unique role in DNA repair during DNA replication in malignant cells.

Interestingly, a recent study showed that RECQ1 silencing in cancer cells induces a cell specific mitotic catastrophe not observed in normal cells [19]. The absence of RECQ1 might promote the accumulation of DNA damage in the M-phase arrested cells due to the deficient G1 and G2 checkpoint functions of cancer cells. These events would then lead to this cell type specific mitotic death. Under normal conditions, the accumulation of DNA damage and mitotic cell death is avoided during the S and G2 phases by the upregulation of different repair enzymes, such as RECQ1. Thus, cancer cells might maintain a greater copy number of DNA repair enzymes to restore DNA damage in a short time since the normal time of cell cycle arrest needed for DNA repair during the S and G2 phases is unavailable due to defects in the checkpoint activity.

Defects in the genes of three of the five human DNA helicases, BLM, WRN and RECQ4, are responsible for distinct genetic disorders associated with cancer predisposition. Moreover, allelic losses or deletion of chromosome 12p12, where the *RECQ1 *gene is located, is a frequent event in a wide range of solid tumors [47-50], and a single-nucleotide polymorphism of the *RECQ1 *gene has been associated with a reduced survival of pancreatic cancer patients [9,10]. In this regard, RecQ helicases might be considered as "tumor suppressors" that prevent neoplastic transformation through the control of chromosomal stability. The fact that RecQ helicases, such as BLM and RECQ1, are upregulated in tumors and provide growth advantage to cancer cells might appear incompatible with the proposed tumor suppression function of these proteins. Recent studies indicated that WRN also supports oncogenic proliferation [17]. Indeed, the expression of several RecQ helicases is also increased upon cellular transformation by EBV and SV40 antigen [41]. A possible explanation for this paradox is that, in somatic cells, DNA repair defects affecting genome integrity due to a RecQ helicase deficiency may lead to cancer predisposition. Conversely, increased RecQ helicase expression might be required in transformed or actively proliferating cells to resolve and repair the elevated load of DNA intermediates that are generated during active replication.

## Conclusions

Taken together, our results indicate that RECQ1 might be considered as a new suitable target for the development of anti-cancer therapies aimed at the elimination of proliferating tumor cells. In this regard, previous studies provided evidence that local and systemic administration of RECQ1-siRNA prevented cancer cell proliferation in vivo in mouse models [18,20]. These studies neglected however human brain glioblastomas, which are, in our opinion, the best type of human tumor where a similar strategy could be applied, given the low amount of RECQ1 present in normal brain cells. Similarly, other DNA repair helicases have been previously proposed as potential targets for anti-tumor therapies mediated by DNA damaging agents or radiation, opening the possibility of using small-molecule inhibitors against DNA helicases as novel chemiotherapeutic agents [51,52].

## Competing interests

The authors declare that they have no competing interests.

## Authors' contributions

RMM, VF, MV, TI, SB, MS, GS, and AV conceived and designed the experiments. RMM, VF, SB, MB, FO, AG, and SB performed the experiments. MV, TI, and MS provided the glioblastoma samples. RMM, VF, SB, GS, and AV analyzed the data. AV and RRM wrote the paper. All authors read and approved the final manuscript.

## Supplementary Material

Additional file 1 **Representative image of the GFAP staining in a peri-lesional (A) and lesional (B) glioma tissue**. The arrows indicate the astrocytes (A), which are positive to the antibody, the oligodentrocytes (O) and the support neurons (N). Click here for file

Additional file 2 **MGMT status in primary glioblastoma highly expressing RECQ1**. A) Box plot representing the count of RECQ1 positive cells expressing in percentage and the methylation status of the MGMT gene from 41 brain primary glioblastomas with staining intensity of 3+ for RECQ1. B) Box plot representing the count of RECQ1 positive cells and MGMT protein expression from 41 brain primary glioblastomas with staining intensity of 3+ for RECQ1. Click here for file

Additional file 3 **MGMT and IDH status in T98G and U-87 glioblastoma cell lines**. A) Gel showing the specific DNA amplicons for unmethylated (uM) and methylated (M) genomic sequences at the 5' of the MGMT gene. MGMT methylation status upon RECQ1 downregulation in different cell lines was also performed using the methylation specific PCR analysis. (MW: DNA marker) B) Representative sequencing chromatograms on IDH1 and IDH2 genes in T98G, U-87 and IMR-90 cells. Dashed boxes indicate the residues R132 and R172 of the *IDH1 *and *IDH2 *genes, respectively. Click here for file
