# Supplementary material for: The human RECQ1 helicase is highly expressed in glioblastoma and plays an important role in tumor cell proliferation
Source: Mol Cancer. 2011 Jul 13;10:83. doi: 10.1186/1476-4598-10-83 (PMC3148559; doi:10.1186/1476-4598-10-83)
Supplement: Additional file 1 — Representative image of the GFAP staining in a peri-lesional (A) and lesional (B) glioma tissue. The arrows indicate the astrocytes (A), which are positive to the antibody, the oligodentrocytes (O) and the support neurons (N). [file 1476-4598-10-83-S1.PDF]

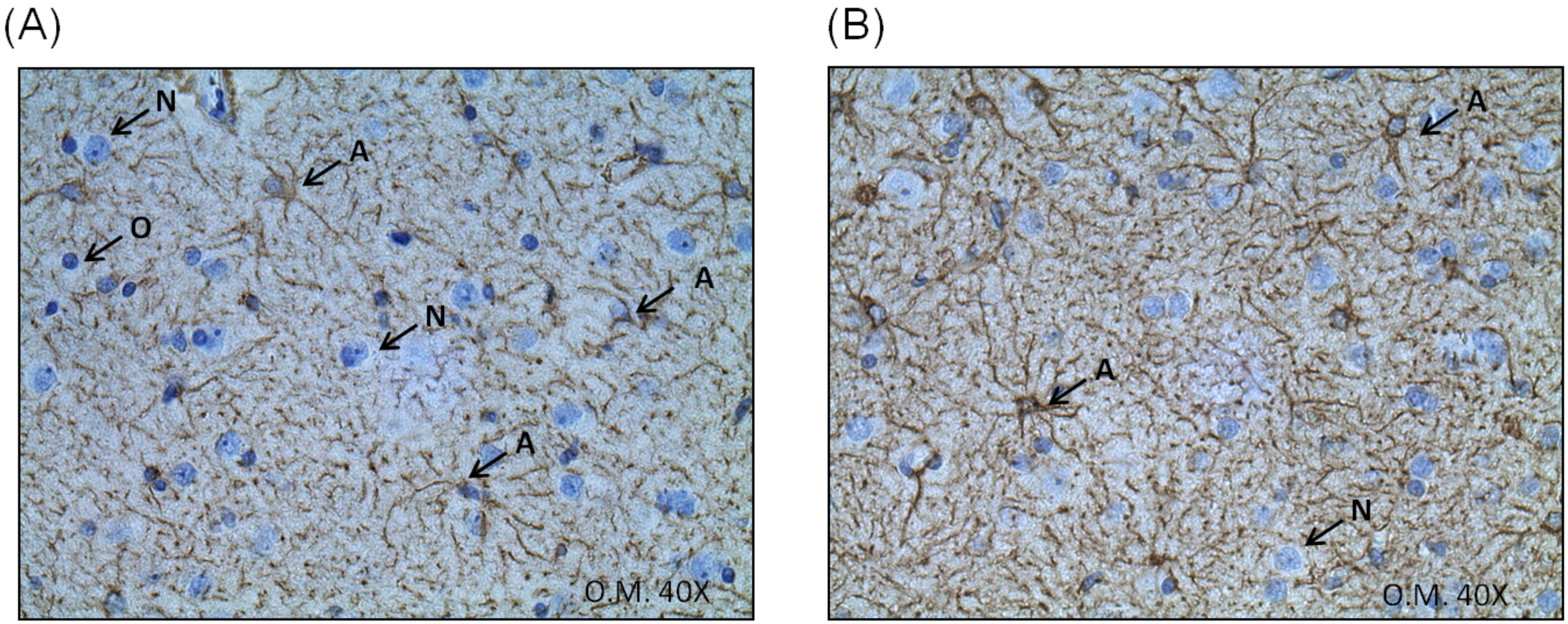

Additional File 1. *Representative image of the GFAP staining in a peri-lesional (A) and lesional (B) glioma tissue*. The arrows indicate the astrocytes (A), which are positive to the antibody, the oligodentrocytes (O) and the support neurons (N).
