# Supplementary material for: The human RECQ1 helicase is highly expressed in glioblastoma and plays an important role in tumor cell proliferation
Source: Mol Cancer. 2011 Jul 13;10:83. doi: 10.1186/1476-4598-10-83 (PMC3148559; doi:10.1186/1476-4598-10-83)
Supplement: Additional file 2 — MGMT status in primary glioblastoma highly expressing RECQ1. A) Box plot representing the count of RECQ1 positive cells expressing in percentage and the methylation status of the MGMT gene from 41 brain primary glioblastomas with staining intensity of 3+ for RECQ1. B) Box plot representing the count of RECQ1 positive cells and MGMT protein expression from 41 brain primary glioblastomas with staining intensity of 3+ for RECQ1. [file 1476-4598-10-83-S2.PDF]

A

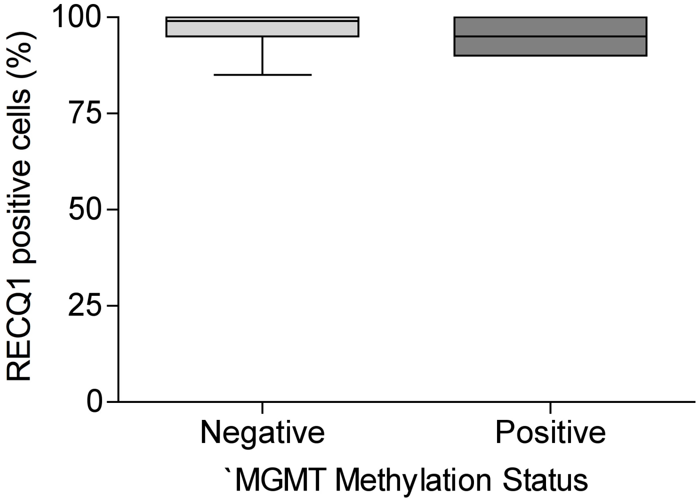

B

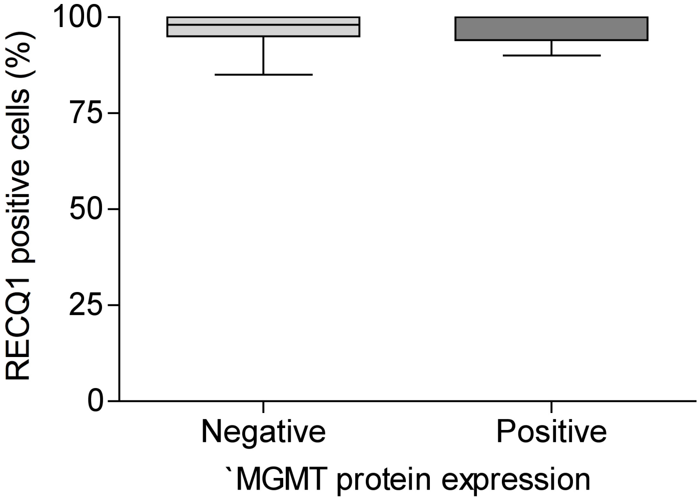

Additional File 2. *MGMT status in primary glioblastoma highly expressing RECQ1.* A) Box plot representing the count of RECQ1 positive cells expressing in percentage and the methylation status of the MGMT gene from 41 brain primary glioblastomas with staining intensity of 3+ for RECQ1. B) Box plot representing the count of RECQ1 positive cells and MGMT protein expression from 41 brain primary glioblastomas with staining intensity of 3+ for RECQ1.
