# Supplementary material for: The human RECQ1 helicase is highly expressed in glioblastoma and plays an important role in tumor cell proliferation
Source: Mol Cancer. 2011 Jul 13;10:83. doi: 10.1186/1476-4598-10-83 (PMC3148559; doi:10.1186/1476-4598-10-83)
Supplement: Additional file 3 — MGMT and IDH status in T98G and U-87 glioblastoma cell lines. A) Gel showing the specific DNA amplicons for unmethylated (uM) and methylated (M) genomic sequences at the 5' of the MGMT gene. MGMT methylation status upon RECQ1 downregulation in different cell lines was also performed using the methylation specific PCR analysis. (MW: DNA marker) B) Representative sequencing chromatograms on IDH1 and IDH2 genes in T98G, U-87 and IMR-90 cells. Dashed boxes indicate the residues R132 and R172 of the IDH1 and IDH2 genes, respectively. [file 1476-4598-10-83-S3.PDF]

A

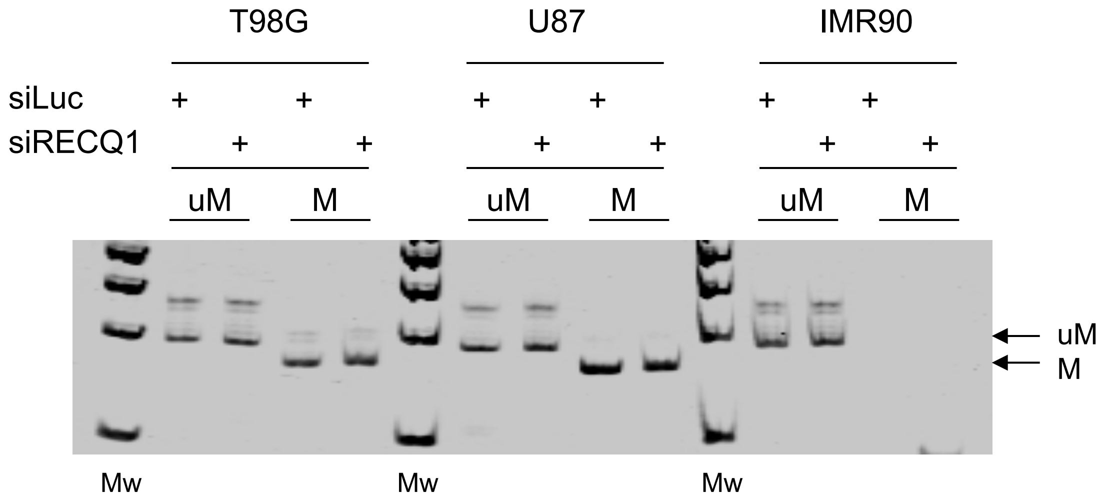

B

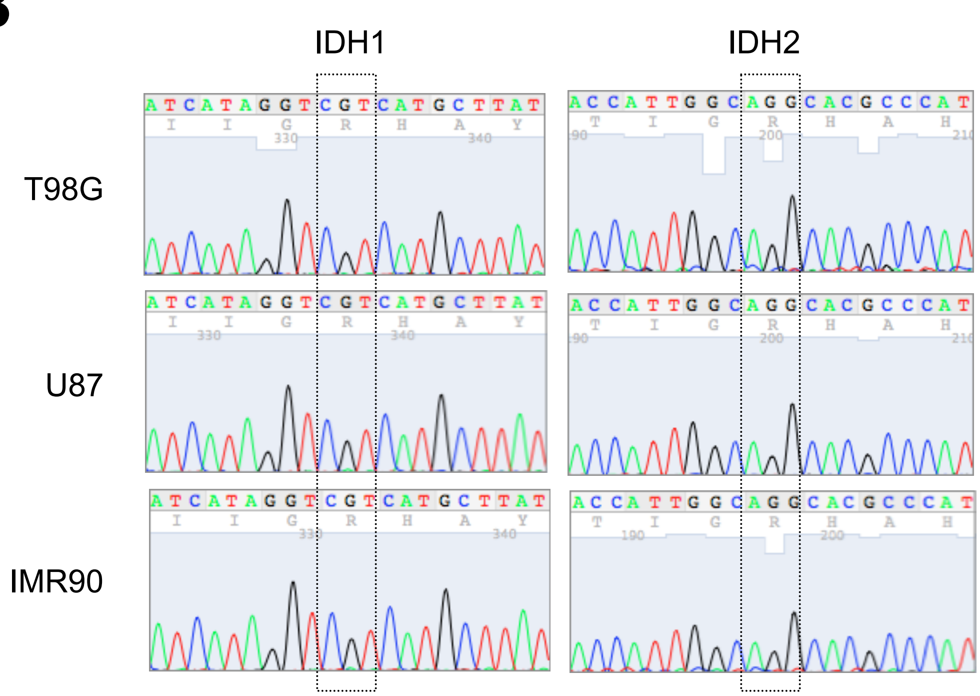

Additional File 3. *MGMT and IDH status in T98G and U-87 glioblastoma cell lines*. A) Gel showing the specific DNA amplicons for unmethylated (uM) and methylated (M) genomic sequences at the 5' of the MGMT gene. MGMT methylation status upon RECQ1 downregulation in different cell lines was also performed using the methylation specific PCR analysis. (MW: DNA marker) B) Representative sequencing chromatograms on IDH1 and IDH2 genes in T98G, U-87 and IMR-90 cells. Dashed boxes indicate the residues R132 and R172 of the *IDH1* and *IDH2* genes, respectively.
